# Acute Peritonitis

**Published:** 1872-11-01

**Authors:** Edwin A. Carpenter

**Affiliations:** Baileyville, Ill.


### ACUTE PERITONITIS.

BY EDWIN A. CARPENTER, M. D., BAILEYVILLE, ILL.

EDITORS MEDICAL EXAMINER:—Conceiving it to be the duty of practitioners of medicine to report the cases in their practice which presents anything unique, either in the management of the cases therapeutically, comparing with text-books, or acknowledged modes of practice, I subjoin an epitomized account of two cases which occurred in my practice two years since.

Case 1. History: Mrs. T. Williams, æt. 24; American; seen May 26th, 1870. Had enjoyed good health until the 23d of the month, when, experiencing acute pain in the umbilical region, she was attacked with diarrhœa. The bowels, judging from the quantity of fecal matter, evacuated their entire contents. She sought the recumbent posture, but obtained no rest from the pain, which rapidly increased. At first, experienced only in the umbilical region, it gradually spread downwards, and became more intense in the hypogastrium. She was the mother of one child six weeks old, which she nursed up to the advent of the disease. Had a chill a few hours prior to my arrival.

Symptoms when first seen: Her appetite is entirely lost. The tongue covered with fur, brown in the center. Lips, dry and cracked. Abdomen somewhat swollen, but would allow of no manipulation, owing to the pain it produced. The respiration is hurried, being nearly thoracic in character, numbering twenty-two per minute. No dullness on percussing the chest. Has frequent desire to pass water, which is voided in small quantity, attended with uneasiness. Face flushed, expression anxious. Pulse, 124–3; severe headache. Treatment: Fomentations to abdomen. ℞ Opium powd. gr. 8. Sub. nit. bismuth, grs. 24. Mix, and divide into eight powders. S. One to be taken every hour until free from pain, or the number of respirations reduced in frequency below their normal standard.

Progress of the case: May 27th. The patient has taken one powder every hour; is in more pain; breathing labored; respirations 21 per minute; pulse 132; bowels quiet; cephalalgia not quite so severe; knees drawn up; pupils slightly contracted. To take strong leaf tea. To have two grains of opium every hour. Sinapism to abdomen, to be left on until redness supervenes. Evening: Abdomen very much distended. Begs for something to quiet the pain. Respirations 20. Pulse 140 beats per minute. Countenance presents great anxiety. Has not passed urine for eight hours, but has desired to do so. Catheterized, getting but a few ounces of urine of an ammoniacal odor. Perspiring profusely. Medicine has been taken regularly.

Left powders containing three grains of opium with one-twelfth grain of calomel, each one of which are to be given every half-hour, until free from pain, or the number of respirations reduced in frequency to ten per minute. Fomentations to be re-applied. 28th: Has taken her medicine every half-hour. Respirations 12. Pulse 135; pupils contracted somewhat. Exquisite pain on slight abdominal pressure. Took nourishment during the night, and frequently complained of the pain she experienced. Directed the continuance of the treatment. Met my esteemed friend, B. T. Buckley, M. D., of Freeport, Ill., in consultation, who fully concurred in the past treatment, and advised its continuance, but thought her beyond recovery. Blister applied; beef tea in suitable quantity.

Evening: Still in *pain.* Has taken her medicine regularly. Blister has not produced any irritation. Catheter. Directed the abdomen to be painted with comp. sol. iodine, to be followed by turpentine stupes. Pulse, 114. Respirations 10. Abdomen distended, seemingly, to its full capacity. At this time a noisome odor pervaded the apartment, rendering it very disagreeable to the ladies in attendance, despite a liberal use of chlorinated soda as a disinfectant. If one brought their hand in contact with her person, the odor would remain for hours, in spite, even, of washing them in a weak solution of carbolic acid. Treatment continued with above additions.

Was called again at 11 P. M. Found her suffering acutely, notwithstanding she took regularly six grains of opium per hour. The pain was more severe in the hypogastrium than elsewhere. Catheter. Left five grains of opium in each powder, *one of which was to be given every half-hour*, until the pain ceased, or the number of respirations reduced to eight per minute. Residing five miles from the patient, I left a goodly number of powders, so in case I was unavoidably detained, she might not be without *opium*. Left a diuretic composed of nitrous ether and fl. ext. digitalis. The same local applications were continued. 29th: Patient free from pain. Her husband gave *ten powders the first hour*, being determined, he said to "ease the pain, if there was *strength* enough in the medicine to do it." One powder every hour thereafter. His statement was coroborated by three ladies of intelligence and good standing in society. Pulse 108. Respiration 10 per minute. Pain *acute* on pressure. She was well under the influence of the drug, but toxic phenomena were not present to an alarming extent. Has taken nourishment. Catheterized.

Directed them to give, not to exceed two powders every hour. Evening. Pulse 107. Respirations 10 Has taken two powders every hour; has taken nourishment. Catheter. Tincture opii was now substituted for the powder, taking one teaspoonful every half-hour, except twice, when she took only one spoonful once per hour. Local applications the same, which produced but little visible effect, save a slight desquamation of the cuticle, caused by the iodine. 30th: Pulse 98. Respiration 10. Gums tender. Not in so much pain. Abdomen less in size. Catheter. Directed the iodine to be omitted, applying only the warm turpentine. One teaspoonful of laudanum per hour. Alcoholic stimulants in small doses. Evening: Patient in less pain. Catheter. June 1st: Increased pain in the hypogastrium. On passing the catheter, the eyes became blocked up with epithelium, which, with agitation, flowed out with about one pint of urine. Diagnosed cystitis as a complication. Directed fomentations to hypogastrium; other treatment continued. Evening: Catheter. A sickening odor emanated from the vagina, which was disinfected with labarques sol. chlorinated soda diluted. One to two drachms of laudanum hourly. Respirations 11. Animal broths in liberal quantities. June 2d: Pain much less severe on pressure. Pulse 86. Abdomen not near so tympanitic. One grain of opium every three hours. Evening: Not in pain unless considerable pressure is used. 3d: Urinated at will. No pain. 4th: Discharged as a regular recipient of visits.

Commentary: The history of this case would lead one to infer that intestinal perforation had occurred. Dr. S. O. Hebershon made an analysis of 500 cases (vide Medical and Surgical Transactions, London, vol. xliii, 1860). From an analysis of the cases he draws the following conclusions: First, It is never idiopathic in its origin, but, when not traumatic, nor due to perforation of viscera, it is dependent either on extension of disease from adjoining viscera, or on certain nutritive changes, or on blood changes, such as occur in pyæmia, albumenuria, etc., or connected with struma, cancer, etc., or lastly, on local hyperœmia, arising from cirrhosis, disease of the heart, etc. The cause must influence the treatment to a greater or less extent. Thus, if from intestinal perforation, keep the bowels quiet until repair has taken place, and, as pain soon exhausts one, *quell the pain;* supporting measures from the onset are advisable to forestall exhaustion.

Case 2d. History: J. Lane, American, æt. 70, wife of a retired farmer. Seen Nov. 11th, 1870. Had enjoyed excellent health up to a few days prior to my being called in; even at her advanced age attending to the domestic duties of her house. Had experienced pains, at first obtuse, but latterly acute in the right inguinal region, which was rapidly diffused over the abdomen, being more intense in the right inguinal region. Her bowels had been regular. The tissues were well nourished, presenting that plumpness of form commonly met with in rural districts, in the persons of young women.

Symptoms when first seen: Her appetite is lost; tongue covered with fur; lips dry; some thirst. Respirations thoracic in character, numbering 23 per minute. Knees drawn up; decubitus dorsal. Physical exploration of chest revealed its contents in a healthy condition. Micturation frequent, and with uneasiness. Countenance presenting that anxious expression so peculiar to acute, abdominal inflammations. Pulse, 128. Some pain in the frontal region of the head. Abdomen swollen and tympanitic, and so very painful I deferred its exploration until she was well under the influence of anodynes.

Treatment: opium powd. ipecacuanha aa. grs. 8. Mix and divide into eight portions. Give two the first hour, one every hour thereafter.

Progress of case: I was called four hours from date of first visit, being told the patient was much worse, *i. e.*, more pain She had taken five powders. Was not nauseated, and said the pain was much more intense. I prepared a solution of morphia, grs. eight to the ounce, of which I gave one drachm the first dose, reducing the number of respirations to eighteen in half an hour, at the end of which time she asked for more, saying it lessened the pain some. I gave one-third of a grain at her solicitation, and my own desire. At the end of fifteen minutes, as the last dose given produced no appreciable effect, I gave her one-third of a grain more, which she thought gave her a little more ease. At the end of the hour, the intellect remaining clear, the pain acute, the number of respirations eighteen per minute, I gave one-third of a grain more, making two grains of morphia administered in one hour.

The second and third hours, she took one and a half grains each hour, reducing the number of respirations to twelve per minute. The pupils were contracted, and the mind somewhat affected, but conversed intelligently when fully aroused. Physical exploration of the abdomen revealed a tumor in the right inguinal region, much the shape and nearly the size of an orange. It was here the pain was most acute. Directed the right inguinal region to be painted with comp. sol. iodine. The abdomen to be covered with flannel, moistened with warm turpentine. Left directions with an intelligent and educated lady to give the solution in half-drachm doses, not oftener than every fifteen minutes, unless the pain increased in severity, and the number of respirations were becoming more frequent. If reduced below twelve to the minute, to suspend its administration until they again arose.

At 10 P. M., the prescription was duplicated. She took one grain per hour since my call in the afternoon. Nov. 12. A. M.: Suffering intensely, owing to a reduction of the last two doses of medicine. The 12th, 13th, and 14th, she took twenty-five grs. of morphine each twenty-four hours, with beef tea and thickened milk for diet. Local applications were continued. The number of respirations were frequently as low as ten, and not to exceed thirteen per minute during this time. The abdomen was still distended, being tympanitic, save over the tumor, which neither enlarged or diminished its size. The pulse had fallen to one hundred beats per minute; varying some each way, but not to any considerable extent. 15th. Pain considerably less, yet suffering greatly upon the least pressure.

The doses of morphine to be reduced one-half, which change was not interfered with, and but little amendment on the part of the patient, until the 18th, at which time the distended abdominal parietes became less voluminous, being yet sore upon pressure of rather an obtuse character. At this time, she disclaimed all knowledge of anything that occurred, save the first day of her treatment. She progressed slowly up to date, 23d, about which time she became free from pain, the tumor presenting the same characteristics as when first examined. It was firm and unyielding to the touch. It was undoubtedly benignant in its nature, being probably a fibrous tumor. Her health has remained good up to this date.

Commentary: The tumor was the primary seat of inflammation, without doubt. She had received no blow, fall or strain. Took plenty of exercise, but did not exert herself beyond her strength. Was not exposed to the inclemency of the weather, in one single instance. The hygienic influences surrounding her were good. In case first, from the best knowledge I have, I do not believe her to have been addicted to the use of opium or alcoholic stimulants. I am satisfied that the opium given was of good quality, and notwithstanding she took, (contrary to directions), fifty grains in one hour, it did not produce even well-marked narcotism. This, to some, may appear incredible, and it is only after a great deal of reflection I have determined to contribute this, being prepared to substantiate what I have averred.

The second case resided but a few steps from my own door, and was frequently seen by me, and though the toxic influence of the drug was marked, yet not to an alarming extent. *Pain is an antidote to opium.* The more intense the pain, the larger the dose of the drug can be administered in safety. Some practitioners are decidedly averse to giving opium in large doses. How they would treat a case of acute diffused peritonitis successfully without it, I know not. The cause must materially influence the treatment. Not long since, bleeding, active purgtion, emetics, a blisters, etc., constituted the treatment. Let us briefly allude to the foregoing modes of treatment. Where dissolution takes place, it is by asthenia. Is bleeding indicated in such a disease? Is purgation the proper mode of treatment in intestinal perforations? Will mercury alone cure the disease? Ramsbotham says (speaking of puerpural peritonitis), "they rarely die, if paralyzed." But he enjoins the use of opium, too. Emetics are depressants, and though exceptionally they might be of value, their general use would bring about what we most fear, depression.

*Quell the pain.* If homœopathic doses will do it, they are large enough. If it requires one or more grains of morphia per hour to do it, they are small enough. So long as there is pain, and the number of respirations are not reduced below eight per minute, there is no danger to be feared from the quantity administered.

